# Long term response on Regorafenib in non-V600E BRAF mutated colon cancer: a case report

**DOI:** 10.1186/s12885-019-5763-5

**Published:** 2019-06-11

**Authors:** Eduard Callebout, Suzane Moura Ribeiro, Stephanie Laurent, Marc De Man, Liesbeth Ferdinande, Kathleen B. M. Claes, Joni Van der Meulen, Karen P. Geboes

**Affiliations:** 1 Department of Gastroenterology, University Hospital Ghent, C Heymanslaan 10, 9000 Ghent, Belgium; 2 Department of Pathology, University Hospital Ghent, C Heymanslaan 10, 9000 Ghent, Belgium; 3 Centre for Medical Genetics, University Hospital Ghent, C Heymanslaan 10, 9000 Ghent, Belgium; 4 Molecular Diagnostics, University Hospital Ghent, C Heymanslaan 10, 9000 Ghent, Belgium

**Keywords:** Case report, Non-V600E BRAF mutation, Colon cancer, Regorafenib, Epithelial to mesenchymal transition

## Abstract

**Background:**

^Non-V600E^ BRAF mutated colorectal cancer (CRC) is a rare disease entity with specific clinical features. These tumors are less likely to have microsatellite instability than CRC with a V600E BRAF mutation and often harbor a KRAS or NRAS mutation. Notably, median overall survival is longer than in wild-type BRAF CRC. Little is known about treatment possibilities in these patients.

**Case presentation:**

We present the case of a 59 year old patient with a rare mutation in BRAF codon 594, who progressed rapidly on all classical therapies but experienced a clear and long lasting response on treatment with Regorafenib.

**Conclusion:**

Little is known about therapies that can be effective in the rare ^non-V600E^ BRAF mutated CRCs. We present a patient who had a definite response to treatment with Regorafenib. There are no predictive markers that define a subset of CRC patients who benefit most from Regorafenib. The specific features of this ^non-V600E^ BRAF mutated CRC may be relevant in the exploration of predictive biomarkers for the efficacy of Regorafenib.

## Background

BRAF is a serine/threonine protein kinase involved in the MAP kinase/ERK-signaling pathway. In this pathway, RAS small guanidine triphosphatase activates the RAF family of proteins (ARAF, BRAF and CRAF). These proteins phosphorylate MEK1/2 proteins, which in turn activate ERKs (extracellular signal-regulated kinases). These regulate a variety of substrates, including multiple transcription factors, thus controlling several key cellular activities. Dysregulation of this pathway induces many elements of tumorigenesis [1, 2].

In CRC it is well known that patients with a BRAF V600E mutation have a poor prognosis. In recent years, extensive molecular testing has led to the diagnosis of other mutations in the BRAF gene. BRAF codon 594 and 596-mutations occur in less than 1–2% of CRC patients, which accounts for 22% of all *BRAF* mutations. Higher incidences have been described and racial differences have been suggested [3, 4].

^Non-V600E^ BRAF mutated tumors differ in molecular and pathological characteristics as well as phenotypically [3, 4]. They are less likely to have microsatellite instability than BRAF V600E mutated CRC and more likely to harbor a *KRAS* or *NRAS* mutation. Median overall survival is longer than in wild type BRAF CRC with a median of 60,7 months demonstrated in a group of 101 patients [4].

Little is known about treatment possibilities in these patients. Some reports with conflicting results have been published on therapy with anti-EGFR antibodies [5, 6].

## Case presentation

A 59-year-old man was diagnosed in July 2014 with a rectal tumor and associated solitary lung metastasis, cT3N1bM1a. He was treated with Folfox-Bevacizumab during 2 months, followed by radiochemotherapy: 25 × 1,8 Gy in combination with oxaliplatin and 5FU. In December 2014, he underwent a total mesorectal excision (TME) together with a video-assisted thoracoscopic resection (VATS) of the lung lesion. The final pathological stage was ypT3N0M1 adenocarcinoma of the rectum and the patient underwent further treatment with Folfox-bevacizumab until the end of March.

In May 2015, at the time of planned restoration of bowel continuity, a relapse was noted in the liver and a resection of segment 4B was performed.

In November 2015, new liver lesions and a peripancreatic mass were found and for the first time a slight elevation of carcinoembryonic antigen (CEA) - 5 μg/L - was noted. Two months after initiation of Folfiri-Bevacizumab, progressive disease (PD) was found on CT scan (with growth of the peripancreatic mass and liver metastases and occurrence of an aortocaval lymph node). The CEA level had risen to 26 μg/L.

In the meantime, molecular analysis was performed and the tumor proved to be *KRAS-NRAS* wild type (WT), *BRAF* mutant with a specific mutation, c.1781A > G (p.(Asp594Gly)) in exon 15 (Next Generation Sequencing (Massively parallel targeted re-sequencing Somatic 1 Multiplicom MASTR assay). Immunohistochemical staining showed no loss of expression of mismatch repair proteins, suggesting microsatellite stability (Antibodies used: Clone ES05 (Novocastra) for MLH1, Clone 6219–1129 (Roche) for MSH2, Clone EP49 (DAKO) for MSH6 and Clone A16–4 (Roche) for PMS2).

Therapy with Folfox-Cetuximab was not successful: there was further progression after 2 months of treatment with occurrence of new liver metastases and a further growth of the peripancreatic lesion and aortocaval lymph nodule. CEA increased to 51 μg/L.

In March 2016, Regorafenib was started at a dose of 160 mg/day (21 days on, 7 days off) while at the same time treatment of the liver metastases with selective internal radiation therapy (SIRT) with Yttrium-90 in combination with stereotactic beam radiation therapy (SBRT) for the para-aortic lymph nodes was planned. Because of a hand-foot skin reaction, treatment with topical corticosteroids and keratolytics was started and a dose modification was made to regorafenib 120 mg/d after 1 treatment cycle. In June 2016, when the treatment with Regorafenib was interrupted in order to proceed to radiotherapy, the CEA level had already dropped to 11 μg/L. SBRT of the para-aortic lymph nodes was administered at a dose of 3 × 8 Gy. CEA was 6 μg/L before selective treatment with Yttrium-90 in the right liver lobe. The patient suffered from bulbitis post radioembolization. In July 2016 a complete remission (CR) was seen in the liver – also in the left liver lobe, which had not been treated with Yttrium-90. CEA had dropped to 5 μg/L.

Regorafenib was stopped in September 2016 after 6 months of treatment.

**Fig. 1:**
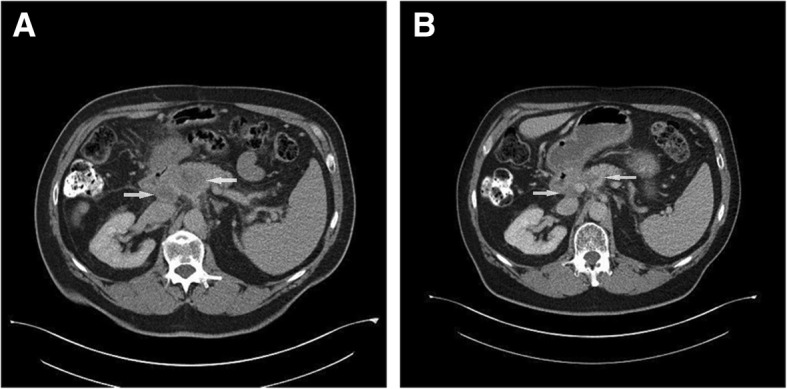
Evolution of periampullary adenopathies on reintroduction of regorafenib. A In january 2017 periampullary adenopathies were seen on follow-up CT-scan. B CT-scan after 6 months of treatment with regorafenib shows a partial response

Re-evaluation at the end of January 2017 showed new lymph nodes in the periampullary region and a rise in CEA level to 12 μg/L. Regorafenib was re-initiated at a dose of 120 mg/d, 3 weeks on, 1 week off. The hand-foot skin reaction was more severe, leading to a personalised treatment schedule - 10 days on/7 days off - in order to increase patient tolerability. Treatment with Regorafenib resulted in normalization of CEA (2 μg/L) and response on CT-scan (Fig. 1) and therapy was stopped in August 2017.

In February 2018, the patient consulted with complaints suggesting gastric outlet obstruction. Endoscopy revealed bulbitis. Chronic inflammation post radioembolization was suspected, but tumor cells were found in the biopsies. CEA had risen to 19 μg/L. A palliative Billroth II resection was performed and tumoral deposits were found both in the duodenum and the antrum.

Regorafenib was reintroduced in April 2018 at a dose of 120 mg/d using the same schedule as in 2017. Re-evaluation in July 2018 showed new adenopathies and a further increase in CEA to 43 μg/L. Shortly thereafter the patient developed jaundice because of biliary obstruction due to a lesion in the liver hilum. Biliary stenting was not possible, but the lesion responded very well to radiotherapy (5 × 4 Gy), resulting in an amelioration of the jaundice. The patient declined further interventions.

**Fig. 2:**
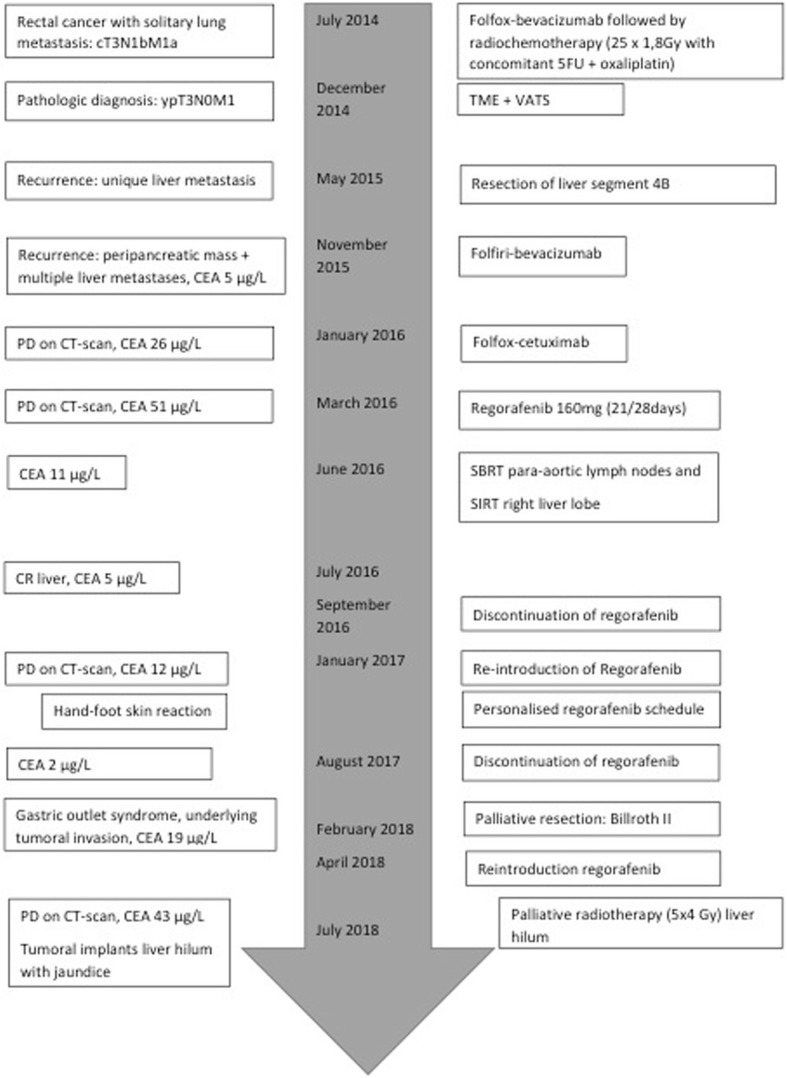
Timeline 2014–2018: Disease characteristics, disease evolution (left column) and therapeutic interventions (right column)

Overall, treatment with Regorafenib with therapeutic breaks resulted in clinical response, both biochemically and radiologically. Disease control was possible during more than 24 months in this patient with a rare *BRAF* mutation. A timeline highlighting the most important disease characteristics, the disease evolution and the therapeutic interventions can be found in Fig. 2.

## Discussion and conclusions

Patients with ^Non-V600E^ BRAF mutated CRC are rare and little is known about treatment possibilities in these patients. Because of the low frequency of these mutations, it will be difficult to investigate the impact of treatment regimens in a prospective (randomized controlled) trial. We present a case of a patient with a specific mutation, c.1781A > G (p.(Asp594Gly)) in exon 15, with a clear response and a long benefit on treatment with Regorafenib.

Regorafenib (BAY 73–4506; Bayer Schering Pharma AG, Berlin, Germany) is an oral small-molecule multikinase inhibitor that is active against several angiogenic receptor tyrosine kinases (RTKs: VEGFR-1, VEGFR-2, VEGFR-3, TIE-2), oncogenic RTKs (c-KIT, RET), stromal RTKs (PDGFR-B, FGFR1), and intracellular signaling kinases (c-RAF/RAF-1, BRAF, BRAFV600E) [7]. A Phase III trial (CORRECT) has demonstrated significant clinical efficacy of Regorafenib in patients with refractory or advanced mCRC [8]. The main effect of Regorafenib on metastatic colorectal cancer in the CORRECT trial seemed to be disease stabilisation, rather than tumor shrinkage. An analysis of the Kaplan–Meier curves for PFS in the CORRECT trial suggests that there may be a distinct subgroup of mCRC patients who are more likely to respond to Regorafenib treatment [7]. Data on the BRAF status of patients were not provided. In vitro activity of Regorafenib in RKO cell lines harboring a V600E BRAF mutation has been shown, but data are scarce and there is no evidence of in vitro activity in other BRAF mutation [9]. Analysis of potential predictive biomarkers for efficacy of Regorafenib is ongoing, but may prove to be difficult because of the nonspecific activity of Regorafenib across a wide range of angiogenic, oncogenic, stromal, and intracellular signaling kinases [7].

The patient we present was a young man with a left-sided (rectal), intermediate grade, microsatellite stable tumor: elements that have often been described in ^Non-V600E^ BRAF mutated CRC patients [4]. On the other hand, the absence of a *KRAS-NRAS* mutation and more importantly the rapid progression upon relapse are less typical features [4].

Our patient had a clear response on Regorafenib. Suggestions about the most interesting pathways to explore as potentially predictive biomarkers for treatment with Regorafenib may be derived from the distinct characteristics of this ^Non-V600E^ BRAF mutated tumor.

Recently, four consensus molecular subtypes (CMS) with specific features have been described in colorectal cancer: CMS1 (MSI Immune): hypermutated, microsatellite unstable tumors with strong immune activation; CMS2 (Canonical): epithelial, chromosomally unstable tumors with marked WNT and MYC signaling activation; CMS3 (Metabolic): epithelial tumors with evident metabolic dysregulation and CMS4 (Mesenchymal): tumors with prominent transforming growth factor β activation, stromal invasion, and angiogenesis [10, 11]. Classical BRAF mutated tumors are most often found in the CMS 1 group [4, 10].

The epithelial to mesenchymal transition (EMT) is an important component of cancer progression. Changes of EMT have been associated with features of advanced disease including metastasis, resistance to chemotherapy, and generation of cancer cells with stem cell-like characteristics [12, 13]. EMT could be impaired in microsatellite instable (MSI) tumors [13]. Similarly, mucinous tumors have been linked to local more than distant recurrence [14]. Thus, the finding that MSI-H and mucinous tumors are more epithelial than mesenchymal seems biologically consistent [14].

Mucinous histology is seen less often in ^Non-V600E^ BRAF mutated CRC and these tumors are usually microsatellite stable [3]. It is possible that CRC with a BRAF mutation occurring at codon 594 specifically represent a mesenchymal phenotype highly dependent on the EMT signature, as can be found in the CMS 4 group [4, 10]. Regorafenib has been shown to target EMT in vitro in colorectal cancer [15]. Furthermore, a greater progression free survival benefit for regorafenib in patients defined as ‘high-risk’ subgroup, according to Marisa molecular subtypes (C4 and C6), corresponding with an upregulation of EMT pathway, has been observed [16–18]. This is one possible explanation for the clear therapeutic advantage seen in our patient that should be further explored.

## Data Availability

Data sharing is not applicable to this article as no datasets were generated or analysed during the current study.

## Abbreviations

BRAF: v-raf murine sarcoma viral oncogene homolog B1
CEA: Carcinoembryonic antigen
CMS: Consensus molecular subtypes
CR: Complete remission
CRC: Colorectal cancer
EMT: Epithelial to mesenchymal transition
ERK: Extracellular signal-regulated kinases
KRAS: Kirsten rat sarcoma 2 viral oncogene homolog
MAP: Mitogen-activated protein
MSI: Microsatellite instable
MYC: v-myc avian myelocytomatosis viral oncogene homolog
NRAS: Neuroblastoma RAS viral oncogene homolog
PD: Progressive disease
RTK: Receptor tyrosine kinase
SBRT: Stereotactic beam radiation therapy
SIRT: Selective internal radiation therapy
TME: Total mesorectal excision
VATS: Video-assisted thoracoscopic surgery
WNT: Wingless-type MMTV integration site family member
WT: Wild type

## References

[1] Ascierto PA, et al. The role of BRAF V600 mutation in melanoma. J Transl Med. 2012;10:85. 10.1186/1479-5876-10-85 PMC3391993 22554099

[2] Sanz-Garcia E (2017). BRAF mutant colorectal cancer: prognosis, treatment, and new perspectives. Ann Oncol.

[3] Cremolini C (2015). BRAF codons 594 and 596 mutations identify a new molecular subtype of metastatic colorectal cancer at favorable prognosis. Ann Oncol.

[4] Jones JC (2017). (Non-V600) BRAF mutations define a clinically distinct molecular subtype of metastatic colorectal Cancer. J Clin Oncol.

[5] Shinozaki E (2017). Clinical significance of BRAF non-V600E mutations on the therapeutic effects of anti-EGFR monoclonal antibody treatment in patients with pretreated metastatic colorectal cancer: the biomarker research for anti-EGFR monoclonal antibodies by comprehensive Cancer genomics (BREAC) study. Br J Cancer.

[6] De Roock W (2010). Effects of KRAS, BRAF, NRAS, and PIK3CA mutations on the efficacy of cetuximab plus chemotherapy in chemotherapy-refractory metastatic colorectal cancer: a retrospective consortium analysis. Lancet Oncol.

[7] Goel G (2018). Evolution of regorafenib from bench to bedside in colorectal cancer: is it an attractive option or merely a “me too” drug?. Cancer Manag Res.

[8] Grothey A (2013). Regorafenib monotherapy for previously treated metastatic colorectal cancer (CORRECT): an international, multicentre, randomised, placebo-controlled, phase 3 trial. Lancet.

[9] Takigawa H (2016). Multikinase inhibitor regorafenib inhibits the growth and metastasis of colon cancer with abundant stroma. Cancer Sci.

[10] Guinney J (2015). The consensus molecular subtypes of colorectal cancer. Nat Med.

[11] Lai E (2018). BRAF-mutant colorectal cancer, a different breed evolving. Expert Rev Mol Diagn.

[12] Polyak K, Weinberg RA (2009). Transitions between epithelial and mesenchymal states: acquisition of malignant and stem cell traits. Nat Rev Cancer.

[13] Pino MS (2010). Epithelial to mesenchymal transition is impaired in Colon Cancer cells with microsatellite instability. Gastroenterology.

[14] Loboda A, et al. EMT is the dominant program in human colon cancer. BMC Med Genet. 2011;4:9. 10.1186/1755-8794-4-9 PMC3032646 21251323

[15] Fan LC (2016). Regorafenib (Stivarga) pharmacologically targets epithelial-mesenchymal transition in colorectal cancer. Oncotarget.

[16] Marisa L (2013). Gene expression classification of colon cancer into molecular subtypes: characterization, validation, and prognostic value. PLoS Med.

[17] Martinelli E (2017). Clinical outcome and molecular characterisation of chemorefractory metastatic colorectal cancer patients with long-term efficacy of regorafenib treatment. ESMO open.

[18] Teufel Michael, Seidel Henrik, Köchert Karl, Meinhardt Gerold, Finn Richard S., Llovet Josep M., Bruix Jordi (2019). Biomarkers Associated With Response to Regorafenib in Patients With Hepatocellular Carcinoma. Gastroenterology.

